# Generation and Characterization of Universal Live-Attenuated Influenza Vaccine Candidates Containing Multiple M2e Epitopes

**DOI:** 10.3390/vaccines8040648

**Published:** 2020-11-03

**Authors:** Tatiana Kotomina, Irina Isakova-Sivak, Ki-Hye Kim, Bo Ryoung Park, Yu-Jin Jung, Youri Lee, Daria Mezhenskaya, Victoria Matyushenko, Sang-Moo Kang, Larisa Rudenko

**Affiliations:** 1 Department of Virology, Institute of Experimental Medicine, Saint Petersburg 197376, Russia; kotomina@iemspb.ru (T.K.); dasmez@iemspb.ru (D.M.); matyshenko@iemspb.ru (V.M.); vaccine@mail.ru (L.R.); 2 Center for Inflammation, Immunity and Infection, Institute for Biomedical Sciences, Georgia State University, Atlanta, GA 30303, USA; kkim39@gsu.edu (K.-H.K.); bpark9@student.gsu.edu (B.R.P.); pharmaco12@gmail.com (Y.-J.J.); youl6248@gmail.com (Y.L.); skang24@gsu.edu (S.-M.K.)

**Keywords:** influenza, universal influenza vaccine, live attenuated influenza vaccine, M2e antigen, recombinant influenza virus, cross-protection, mouse model, B-cell immunity, T-cell immunity, innate immunity

## Abstract

Influenza viruses constantly evolve, reducing the overall protective effect of routine vaccination campaigns. Many different strategies are being explored to design universal influenza vaccines capable of protecting against evolutionary diverged viruses. The ectodomain of influenza A M2e protein (M2e) is among the most promising targets for universal vaccine design. Here, we generated two recombinant live attenuated influenza vaccines (LAIVs) expressing additional four M2e tandem repeats (4M2e) from the N-terminus of the viral hemagglutinin (HA) protein, in an attempt to enhance the M2e-mediated cross-protection. The recombinant H1N1+4M2e and H3N2+4M2e viruses retained growth characteristics attributable to traditional LAIV viruses and induced robust influenza-specific antibody responses in BALB/c mice, although M2e-specific antibodies were raised only after two-dose vaccination with LAIV+4M2e viruses. Mice immunized with either LAIV or LAIV+4M2e viruses were fully protected against a panel of heterologous influenza challenge viruses suggesting that antibody and cell-mediated immunity contributed to the protection. The protective role of the M2e-specific antibody was seen in passive serum transfer experiments, where enhancement in the survival rates between classical LAIV and chimeric H3N2+4M2e LAIV was demonstrated for H3N2 and H5N1 heterologous challenge viruses. Overall, the results of our study suggest that M2e-specific antibodies induced by recombinant LAIV+4M2e in addition to cellular immunity by LAIV play an important role in conferring protection against heterologous viruses.

## 1. Introduction

Influenza is a highly contagious pathogen possessing a serious threat to worldwide human population [1]. The influenza viruses have been studied intensively, but still a lot of challenges remain regarding the disease control and prevention [2]. The main obstacle for controlling influenza outbreaks is that the influenza virus tends to evolve and accumulate genetic changes to escape anti-influenza population immunity, thus generating novel epidemic and pandemic strains. One of the processes termed «antigenic drift» indicates the point mutation in genes encoding surface proteins hemagglutinin (HA) and neuraminidase (NA) of influenza strains circulating in human population. Another evolutional mechanism, termed «antigenic shift», results in introduction of novel surface protein variants specific to non-human species, such as avian or swine, in human circulating influenza viruses. Seasonal routine immunization programs are proposed as a cost-effective tool of preventive medicine measures against influenza. However, current influenza virus vaccines are effective only when the predicted vaccine strains and circulating viruses are well-matched. The current strategy of influenza vaccination does not prevent the pandemic outbreaks, and protection efficacy is reduced or ineffective if mutant strains emerge.

Currently licensed vaccine strategies are developed to induce antibody-specific responses directed against predicted influenza strains. Mismatches between vaccine and circulating strains or the emergence of a new pandemic virus can reduce the vaccine effectiveness and greatly limit the effectiveness of seasonal vaccination campaigns. The development of a universal vaccine which provides cross-reactive and long-lasting immune responses against divergent influenza strains can solve these problems [3,4,5]. One of the promising targets for designing broadly protective influenza vaccines is an extracellular domain of matrix 2 protein (M2e), since it is well conserved among human, avian and swine influenza A viruses [6,7]. However, M2e itself is known to be a very poor immunogen due to its small size, low copy numbers of M2 in the virion, and the possible shielding effect of larger surface proteins of influenza A virus [8,9,10]. Only a minority of people infected with influenza exhibited anti-M2e antibodies [11]. Even live influenza virus infection that generates strong humoral and cellular immunity is not effective in inducing M2e-specific antibodies in a mouse model [12,13]. Therefore, previous studies have focused on increasing the immunogenicity of M2e using a variety of carrier vehicles and/or in combination with various adjuvants (reviewed in [7,14]).

One of the most promising platforms for presenting M2e epitopes to the immune system is the use of virus-like particles that prime both humoral and cellular immunity against influenza [15,16]. Another possible vehicle for the delivery of the M2e epitopes to the immune system, which is capable of inducing both antibody and T-cell immune responses, is live attenuated influenza vaccine (LAIV) virus. An intranasal delivery of LAIV virus offers an easy accessible route to the immune system, inducing robust mucosal antibody responses, as well as T-cell immunity [17,18]. This platform was successfully used for designing universal influenza vaccines based on another conserved viral protein—HA stalk domain—yielding promising results in animal models [19,20,21].

In this study, we designed new universal influenza vaccine candidates by insertion of four M2e tandem repeats into HA molecules of H1N1 and H3N2 LAIV reassortant viruses based on the licensed cold-adapted A/Leningrad/134/17/57 backbone and tested their safety, immunogenicity and cross-protective efficacy in a mouse model, with a special attention to identifying of the possible immune mechanisms of protection. The four M2e repeats were used to cover a broader M2e variants of human, swine and avian influenza A viruses.

## 2. Materials and Methods

### 2.1. Viruses, Cells, Proteins, Antibodies

Wild-type human influenza viruses A/South Africa/3626/2013 (H1N1) and A/Switzerland/9715293/2013 (H3N2) were obtained from NIBSC (London, UK) and CDC (Atlanta, GA, USA), respectively. Mouse-adapted viruses A/Philippines/2/82 (H3N2) and a PR8-based reassortant A/Vietnam/1203/04-PR8 (rgH5N1) were used as previously described [13]. Viruses were cultured in eggs at 37 °C, allantoic fluid was clarified by low-speed centrifugation and stored at −70 °C in single-use aliquots.

Madin–Darby Canine Kidney (MDCK) and Vero epithelium cell lines were obtained from ATCC (American Type Culture Collection, Manassas, VA, USA). MDCK cells were maintained in growth Dulbecco’s modified Eagle medium (DMEM) (Thermo Fisher Scientific, Waltham, MA, USA) containing 10% fetal bovine serum (FBS) (Thermo Fisher Scientific, USA) and 1x antibiotic-antimycotic (Thermo Fisher Scientific, USA) at 37 °C in the atmosphere of 5% CO_2_. Vero cells were growing in OptiPRO SFM media (Thermo Fisher Scientific, USA) supplemented with antibiotic-antimycotic and GlutaMAX (Thermo Fisher Scientific, USA) at the same conditions.

An M2e peptide sequence (SLLTEVETPIRNEWGSRSN) highly conserved among human influenza A viruses (hM2e) was chemically synthesized (GenScript, Piscataway, NJ, USA). The 14C2 monoclonal anti-M2e antibody was purchased from Abcam (Cambridge, UK).

### 2.2. Generation of Recombinant LAIV Viruses

**Figure 1:**
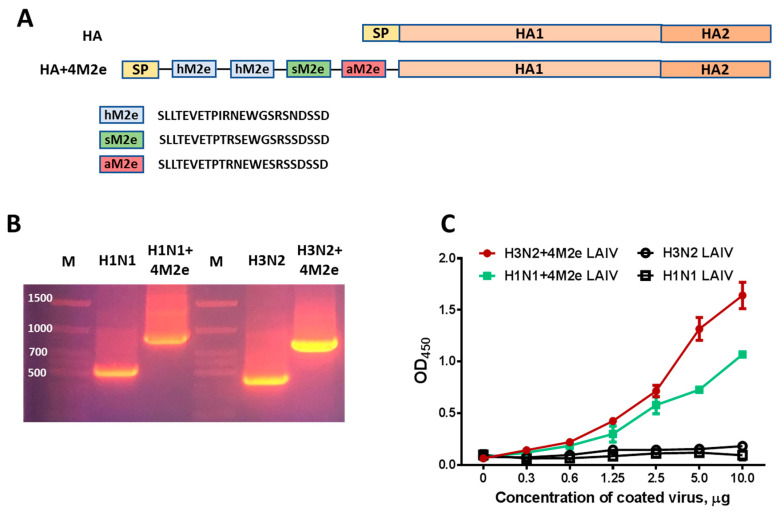
Chimeric live attenuated influenza vaccine (LAIV)+4M2e LAIV viruses generated in this study. (**A**) Schematic representation of the chimeric hemagglutinin molecule containing four M2e tandem repeats inserted between the signal peptide (SP) and HA1 subunit. (**B**) RT-PCR product of amplification of hemagglutinin (HA) gene fragments of the recombinant and natural H1N1 and H3N2 influenza viruses. The H1N1 HA gene was amplified with F1 and R406 primers; the H3N2 HA gene was amplified with F1 and R322 primers. M: DNA molecular weight marker. (**C**) Expression of M2e epitopes at the surface of the recombinant and natural H1N1 and H3N2 influenza viruses.

Gene sequence coding for the four conserved epitopes derived from the M2 protein ectodomain specific to human, swine and avian-type influenza viruses separated by AAAPGAA or AAAGGAA linkers were chemically synthetized (GenScript, Piscataway, NJ, USA) as previously described [22]. The 4M2e cassette was cloned between the signal peptide domain and HA1 subunit of a hemagglutinin (HA) molecule of A/South Africa/3626/2013 (SA) or A/Switzerland/9715293/2013 (SW) virus (Figure 1A). Of note, the Cys17 and Cys19 positions of natural M2e were substituted by Ser17 and Ser19 to prevent M2e intermolecular aggregation. Such substitutions are known to have no effect on immunogenic properties of M2e [23] or M2e expression level [24].

The chimeric HA+4M2e genes were then inserted into a dual-promoter plasmid vector for influenza reverse genetics (pCIPolISapIT). The recombinant LAIV+4M2e viruses, along with their matched control LAIV reassortants were generated on the A/Leningrad/134/17/57 (Len/17) backbone [25]. Vaccine viruses were rescued by electroporating Vero cells with the desired set of eight plasmids using Neon Transfection System, according to the manufacturer’s instructions (Invitrogen, Carlsbad, CA, USA). Rescued viruses were amplified in eggs at 33 °C and their full genomes were sequenced by Sanger sequencing to confirm their identities of inserted genes. For immunological assays, the classical H1N1 and H3N2 LAIV strains were amplified in eggs, concentrated on sucrose gradient and stored in aliquots at −70 °C, and 200 ng of purified viruses were coated per well of ELISA 96-well plates.

### 2.3. In Vitro Studies

The insertion of the 4M2e tandem repeats into recombinant virus HA genes was initially confirmed by Reverse transcription polymerase chain reaction (RT-PCR), for which viral RNA was extracted using QIAamp Viral RNA Mini Kit (QIAGEN, Hilden, Germany), followed by one-step RT-PCR (SuperScrip One-Step RT-PCR System with Platinum™ Taq, Invitrogen, USA) with a universal HA-F1 forward primer (AGCAAA AGCAGGGG) and R406 (TGGGGAATATCTCAAACCTT) or R322 (TGCTGCGTTCAACAAAAAGGTC) reverse primer for H1N1 and H3N2 LAIV viruses, respectively.

Expression of the M2e epitopes on the surface of recombinant LAIV+4M2e viruses was assessed by ELISA. For this, Corning^®^ (New York, NY, USA) ELISA 96-well microplates were coated with sucrose gradient-purified chimeric LAIV+4M2e influenza viruses, along with their classical LAIV counterparts in duplicates at the following concentrations: 10, 5, 2.5, 1.2, 0.6, 0.3 µg per well overnight at 4 °C. Total protein concentration of purified LAIV viruses was determined by DC Protein Assay Kit (BIO-RAD, Hercules, CA, USA). Then, plates were washed three times with 0.1% phosphate buffered saline with Tween 20 (PBS-T) solution and blocked with 1% BSA. After 3 additional washes with PBS-T, the monoclonal antibody 14C2 was added, followed by incubation for 1 h at 37 °C. The binding ratio to surface-adsorbed antigens was determined after incubation with secondary goat anti-mouse immunoglobulin G (IgG) conjugated to horse radish peroxidase (HRP) (Abcam, USA, ab205719). The results were assayed using 3,3′,5,5′-tetramentylbenzidine 1-Step Ultra TMB-ELISA substrate (Thermo, USA) at 450 nm using Bio-Tek ELISA plate reader.

LAIV viruses used in this study were assessed by their infectivity in eggs and MDCK cells. Temperature-sensitive (*ts*) and cold-adapted (*ca*) phenotypes were determined by virus titration at different temperatures in eggs: 38 °C compared to 33 °C for *ts* phenotype and 26 °C compared to 33 °C for *ca* phenotype. Eggs inoculated with 10-fold virus dilutions were incubated for either 48 h (for 33 °C and 38 °C) or 6 days (for 26 °C). In addition, growth characteristics of the LAIV viruses were analyzed in MDCK cells. For this, cell monolayers were infected with studied viruses at a multiplicity of infection (MOI) 0.01 in triplicates and 150 µL of the media were collected every 12 h and stored at −70 °C prior to titration by TCID_50_. Virus titers in eggs and MDCK cells were calculated by Reed and Muench method [26] and expressed in log_10_EID_50_/mL and log_10_TCID_50_/mL, respectively. A virus was considered to be temperature sensitive (*ts* phenotype) if the infectious titers at 33 °C were at least 5.0 log_10_EID_50_ higher than at 38 °C. A virus was considered to be cold adapted (*ca* phenotype) if the infectious titers at 26 °C were not more than 3.0 log_10_EID_50_ lower than at 33 °C.

### 2.4. Replication of LAIV Viruses in Mouse Respiratory Tract

Female 6–8 weeks old BALB/c mice were purchased in the Jackson Laboratories (Sacramento, CA, USA). Groups of 4 mice were infected with H1N1 and H3N2 chimeric LAIV+4M2e viruses, as well as with their classical LAIV analogs, with a volume of 50 µL of virus suspension containing 10^6^ or 10^7^ EID_50_ by the intranasal (i.n.) route. Nasal turbinates and lungs were collected at 3 days post infection (dpi) and stored frozen at −70 °C until used for homogenization. Tissue homogenates were prepared using frost glass slides in 1 mL of sterile phosphate buffered saline (PBS) containing antibiotic-antimycotic (Invitrogen, UK) and the clarified supernatants were used to determine virus titers by limiting dilutions in eggs as described above. The limit of detection was 1.2 log_10_EID_50_/mL.

### 2.5. Direct Protection against Heterologous and Heterosubtypic Challenge

Groups of female BALB/c mice (6–8 weeks old) were immunized intranasally with chimeric H3N2+4M2e LAIV and classical H3N2 LAIV viruses at a dose of 10^7^ EID_50_, in a volume 50 µL. Three weeks later, mice received a second booster dose of the same vaccine. Blood samples were collected 3 weeks after each dose via retro-orbital sinus to assess antibody immune responses in ELISA using either sucrose-purified LAIV virus or human M2e peptide as antigens. ELISAs were performed as described above, with 200 ng of total protein coated per each well of ELISA 96-well plates. Three weeks after the booster dose immunized mice at the age of 12–14 weeks old were challenged either with A/Philippines/2/82 (500 LD_50_) or A/Vietnam/1203/04-PR8 (40 LD_50_) virus. All mice were then monitored daily for body weight loss and survival rate during 2 weeks.

### 2.6. Indirect Protection (In Vivo Protection Assay of Immune Sera)

To determine whether the immune sera from mice immunized with the studied LAIV or LAIV+4M2e vaccine candidates would contribute to cross-protection against a different subtype of influenza A viruses, we carried out an in vivo protection assay as described in [27]. Briefly, sera from immunized or naïve mice were mixed with PBS at a 1:1 ratio and heat-inactivated at 56 °C for 1 h. Then the inactivated sera were mixed 1:1 with A/Philippines/2/82 (5 LD_50_) or A/Vietnam/1203/04-PR8 (5 LD_50_) virus and incubated at room temperature for 30 min. Groups of 5–6 naive BALB/c mice were infected intranasally with a mixture of virus and sera at a volume 50 µL and were monitored for their survival rates and weight loss as for two weeks.

### 2.7. Comparing Immunogenicity and Cross-Protection of Homologous and Heterologous Prime-Boost Vaccination Regimens

In this experiment, different prime-boost vaccination strategies were performed. Groups of five mice (6–8 weeks old) were immunized intranasally either with two doses of H1N1+4M2e LAIV or with one dose of H1N1+4M2e LAIV, followed by the second dose of H3N2+4M2e LAIV, at a three-week interval. Control groups were immunized with the corresponding LAIVs, using the same combinations. The H1N1 LAIVs were administered at a dose 10^6^ EID_50_, while H3N2 LAIVs were inoculated at dose 10^7^ EID_50_. The placebo group received PBS. Blood samples were collected three weeks after each dose for the assessment of antibody immune responses. The whole virus-specific and M2e-specific serum IgG antibody, as well as IgG1 and IgG2a isotypes were determined in ELISA as described above, except that the secondary HRP-conjugated antibodies were different: IgG, IgG1, IgG2a (Southern Biotech, Birmingham, AL, USA).

To assess the cross-protection of the different vaccination regimens, immunized mice at the age of 23–25 weeks old were challenged with rgH5N1 (20 LD_50_) virus four months after boost vaccine dose. Five days after infection, mice were sacrificed; blood, bronchoalveolar lavage (BAL) and different tissues (lungs, spleen, MLN) were collected to estimate antibody- and cell-mediated immunity. In addition, lung viral titers post-challenge were determined as described above.

Antibody responses in mediastinal lymph nodes (MLN) and spleen after infection with heterologous challenge virus were measured as described earlier [28]. Briefly, MLN and spleen cells collected at day 5 post infection were cultured in the presence of hM2e peptide or inactivated H1N1 LAIV or H3N2 LAIV whole virus antigens for 5 days at 37 °C. The concentrations of IgG antibody were measured by ELISA. 

To determine cell-mediated immune responses, bronchoalveolar lavage (BAL) fluids and lung tissues were collected at day 5 post infection. BAL cells were harvested by infusing 1 mL of PBS into the trachea using a catheter (Exelint International Co., Los Angeles, CA, USA) as described in [28]. The lung tissues were homogenized and spun on 44%/67% Percoll gradients at 2800 rpm for 15 min. The lung cells were collected from the layer between 44% and 67%. Cellular phenotypes including innate or effector T cells secreting cytokines were analyzed by flow cytometry as described in [15]. In brief, the following cell surface marker antibodies were used to stain single cell preparations: fluorophore-labeled antibodies specific for anti-mouse CD45 (clone 30-F11), CD11b (clone M1/70), CD11c (clone N418), F4/80 (clone BM8), Ly6c (clone HK1.4), MHC class II (clone M5/114.15.2), CD3 (clone 17A2), CD4 (clone GK1.5), CD8 (clone 53–6.7). The cellular phenotypes were gated as follows: total dendritic cells (DCs), CD45^+^F4/80^−^CD11c^+^ MHCII^high^; monocyte-derived macrophages, CD45^+^CD11b^+^Ly6c^high^F4/80^+^; neutrophils, CD45^+^CD11b^+^Ly6c^+^F4/80^−^. Intracellular cytokine staining (ICS) was performed by using antibodies for intracellular cytokines and surface markers including IFN-γ (eBioscience), TNF-α (BioLegend), CD45, CD3, CD4, CD8 (BD Biosciences). All samples were processed on a Becton-Dickinson LSR-II/Fortessa flow cytometer (BD, San Diego, CA, USA) and analyzed using flowjo software (FlowJo V10, Tree Star, Inc., Ashland, OR, USA).

### 2.8. Hemagglutination Inhibition (HAI) Assay

Immune sera were treated with receptor destroying enzymes (RDE, Sigma Aldrich, St. Louis, MO, USA) at 1:3 ration (sera:RDE) and incubated for 16 h at 37 °C as previously described [28]. The RDE-treated serum samples were inactivated at 56 °C for 30 min, then serially 2-fold diluted, and incubated with 4 HA units of H1N1 LAIV, H3N2 LAIV, rgH5N1, and H3N2 (Phil) viruses for 30 min, then admixed with 0.5% chicken red blood cells (RBC, Lampire Biological Laboratories, Pipersville, PA, USA).

### 2.9. Ethical Statement

All studies involving mice were followed the accepted principles of Institutional Animal Care and Use Committee (IACUC) guidelines in Georgia State University (approval code A18001). Experimental procedures included the intranasal administration of vaccine candidates or/and challenge, and bleeding was performed under isoflurane anesthesia to minimize suffering. Once clinical signs of influenza infection appeared critical in mice, the humane euthanasia using overdose isoflurane inhalation was performed.

### 2.10. Statistical Analysis

The data were collected and analyzed using GraphPad Prism 5 (GraphPad Software, Inc., San Diego, CA, USA). The statistical significance of the difference between viral titers in organs of mice was determined by the Mann–Whitney U-test. The statistical significance of immunogenicity outcomes was determined by one-way or two-way ANOVA followed by a Tukey’s multiple comparison test. *p* values of <0.05 were considered significant.

## 3. Results

### 3.1. Expression of M2e Epitopes by LAIV+4M2e Chimeric Vaccines

The rescued H1N1+4M2e and H3N2+4M2e recombinant LAIV viruses indeed carried HA RNA molecules which were approximately 360 nucleotides longer than the HA genes of corresponding classical LAIV viruses (Figure 1B). To confirm that the inserted four M2e tandem repeats are expressed on the surface of the recombinant LAIV+4M2e viruses, we performed ELISA, where sucrose gradient-purified LAIV viruses were used as coating antigens and M2e-specific 14C2 was used as a primary antibody. Both H1N1 and H3N2 recombinant LAIV+4M2e viruses were able to bind 14C2 antibody, whereas their classical analogs did not react with this antibody, suggesting that the additional M2e epitopes are indeed presented at the surface of the viral particles (Figure 1C). The relatively lower levels in M2e-specific 14C2 monoclonal antibody reactivity of H1N1+4M2e LAIV compared to those of H3N2+4M2e LAIV might be due to the differences in HA protein abundance relative to total viral protein between H1N1 and H3N2 LAIV strains [29].

### 3.2. Replication of Chimeric LAIV+4M2e and Their Classical LAIV Counterparts In Vitro and In Vivo

**Figure 2:**
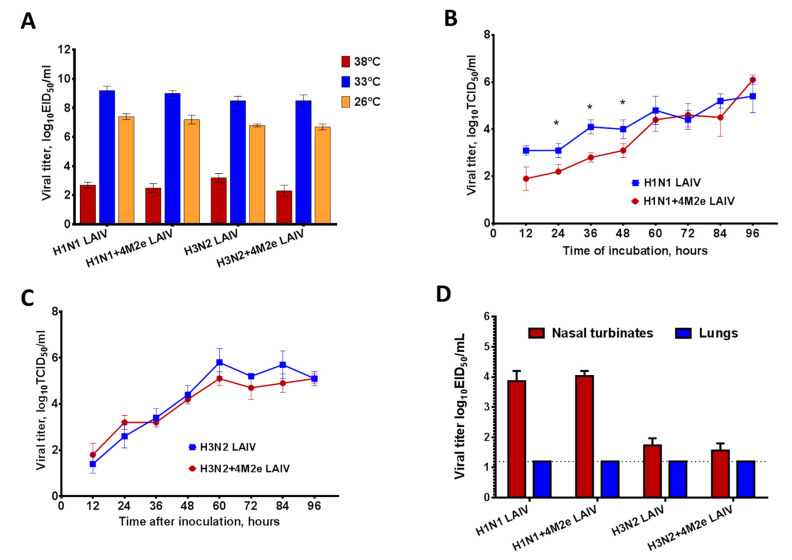
Replication of LAIV reassortants in vitro and in vivo. (**A**) Viral titers in eggs at different incubation temperatures. (**B**) Kinetics of virus growth of H1N1 LAIVs in Madin–Darby Canine Kidney (MDCK) cells at MOI 0.01. (**C**) Kinetics of virus growth of H3N2 LAIVs in MDCK cells at MOI 0.01. (**D**) Replication of LAIV viruses in mouse respiratory tract at day 3 post inoculation (H1N1 LAIVs and H3N2 LAIVs were administered at doses 10^6^ EID_50_ and 10^7^ EID_50_, respectively). Data were compared with the Mann–Whitney U-test (* *p* < 0.05),

We further compared the temperature-sensitive (ts), cold-adapted (ca) and attenuated (att) phenotypes of the chimeric LAIV viruses expressing four M2e tandem repeats with corresponding classical LAIV strains. As shown in Figure 2, the insertion of additional M2e epitopes into HA molecule of the vaccine viruses did not alter their replicative characteristics. All studied vaccine candidates replicated equally well at optimal temperature 33 °C, as well as at lower temperature 26 °C, whereas their replication at elevated temperature 38 °C was impaired, suggesting that the chimeric viruses maintained ts/ca phenotypes similar to the classical LAIV strains (Figure 2A).

The 4M2e inserts did not have a negative impact on the growth kinetics of the H1N1 and H3N2 LAIV strains on MDCK cells. Although the H1N1+4M2e LAIV grew slower during the first 48 h than corresponding H1N1 LAIV, infectious titers of both H1N1 LAIVs became comparable at 60 h.p.i and increased up to 96 h.p.i. (Figure 2B). There was no difference at any timepoint for the infectious titers of the H3N2+4M2e and H3N2 LAIVs (Figure 2C). All LAIVs tested reached similar peak titers ~6.0 log_10_TCID_50_/mL.

The insertion of four M2e tandem repeats also did not affect LAIV’s ability to replicate in mouse respiratory tract: no differences were detected in viral titers in nasal turbinates or lung tissues between LAIV+4M2e virus and the corresponding LAIV strain (Figure 2D). Importantly, the H3N2 LAIVs did not produce infectious virus in mouse respiratory tract when administered at a dose 10^6^ EID_50_ (data not shown), whereas replication in nasal turbinate tissues were seen when mice were infected with a dose 10^7^ EID_50_. Therefore, the dose 10^7^ EID_50_ was used in our further immunogenicity and protection analyses. It is notable that both LAIV+4M2e vaccine candidates recapitulated the attenuated phenotype of the related LAIV strains: there was no replication of the vaccine viruses in mouse lungs, whereas significant titers were seen in the upper respiratory tract (Figure 2D).

### 3.3. Immunogenicity of the Recombinant LAIV+4M2e Viruses

**Figure 3:**
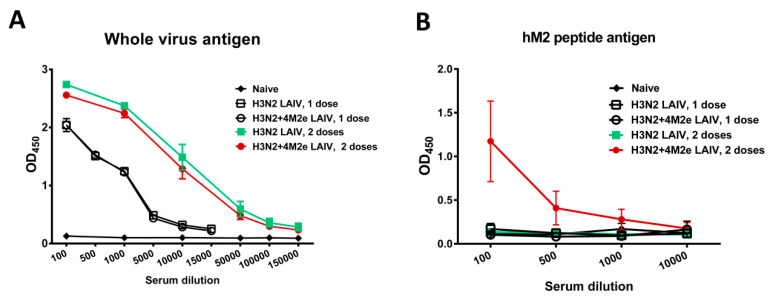
Serum IgG antibody responses to whole influenza virus antigen (**A**) and to human M2e peptide (**B**).

Mice were intranasally immunized with 1 × 10^7^ EID_50_ of H3N2 LAIV+4M2e and the corresponding classical H3N2 LAIV strain, twice with a 3-week interval. Serum antibody levels were assessed after each vaccine dose by ELISA, both against whole virus antigen and human M2e peptide. Both vaccines were immunogenic and induced high levels of virus-specific IgG, which were significantly boosted after the second immunization (Figure 3A). Importantly, significant levels of M2e-binding antibody were induced only by the LAIV+4M2e vaccine candidate, which required a booster vaccine dose (Figure 3B). Since the classical LAIV strain did not induce detectable M2e-binding IgG antibody, the expression of additional M2e epitopes on the surface of viral particle was responsible for the induction of such antibodies.

### 3.4. Protection against Heterologous and Heterosubtypic Viruses

To find out if the insertion of four M2e tandem repeats could improve the cross-protective potential of the H3N2 LAIV virus, immunized mice were challenged with heterologous H3N2 virus (A/Philippines) and a heterosubtypic virus H5N1 virus (A/Vietnam). Both vaccines equally well protected animals from H3N2 (A/Philippines) and rgH5N1 (reassortant A/Vietnam) challenge viruses (data not shown), suggesting that antibody and cell-mediated immunity by prime boost with H3N2 LAIV virus vaccines contributed to the protection.

**Figure 4:**
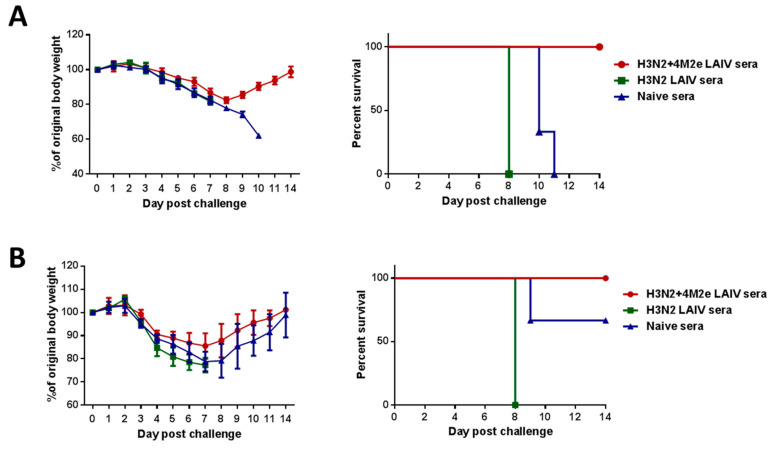
Passive protection by immune sera against challenge with heterologous A/Philippines/2/82 (H3N2) virus (**A**) and A/Vietnam/1203/04-PR8 (rgH5N1) virus (**B**). The left panel shows dynamics of body weight change; the right panel shows survival rates.

To find out if the induced M2e antibody could contribute to the enhanced protection of immunized animals against heterologous influenza virus infection in the absence of T-cell-based immunity, we conducted an in vivo protection study using the same H3N2 and rgH5N1 heterologous strains. For this, immune sera from mice after two doses of either H3N2 LAIV or H3N2 LAIV+4M2e or PBS were mixed with each challenge virus and administered intranasally to naïve mice. As shown in Figure 4, sera from mice vaccinated with H3N2 LAIV+4M2e vaccine afforded better protection against death and weight loss than sera from corresponding classical H3N2 LAIV, suggesting that the H3N2+4M2e LAIV virus induced more broadly protective antibodies, which were most probably specific to the M2e epitope.

### 3.5. Assessment of Antibody Immune Responses After Homologous and Heterologous Prime-Boost Immunization with LAIV+4M2e Universal Vaccine Candidates

**Figure 5:**
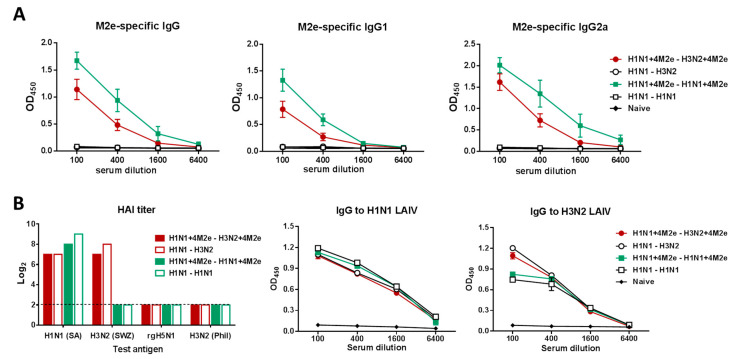
M2e-specific and influenza-specific antibody immune response after prime-boost vaccination. (**A**) M2e-specific serum IgG (left graph), IgG1 (middle graph) and IgG2a (right graph) antibodies were measured in ELISA using hM2e protein as a coating antigen. (**B**) Influenza virus-specific antibody responses. HAI antibodies were measured against a panel of influenza viruses using mouse sera after prime and boost vaccinations (left graph). The dotted line indicates the limit of antibody detection. Influenza-specific serum IgG antibodies were measured in ELISA against whole virus antigens H1N1 LAIV (middle graph) and H3N2 LAIV (right graph).

To better understand the mechanisms of protection afforded by the recombinant LAIV viruses carrying additional M2e epitopes on their surface, we performed further studies of immunizing additional groups of BALB/c mice with prime dose of H1N1+4M2e LAIV, followed by the boost dose of H3N2+4M2e LAIV, or homologous prime boost doses of H1N1+4M2e LAIV. Control groups were immunized with the corresponding LAIVs. These regimens were chosen since H1N1 LAIVs replicated better in the upper respiratory tract than H3N2 LAIVs (Figure 2D). Furthermore, the heterologous prime-boost vaccination was explored to reduce the immunodominance of the HA epitopes. Since the two chimeric LAIVs shared only 4M2e repeats but differed significantly by the HA source, heterologous prime-boost immunization was supposed to boost antibodies targeting M2e but not HA. As expected, serum M2e-binding IgG antibodies were induced only by the LAIVs expressing additional 4M2e tandem repeats, whereas classical LAIVs did not induce M2e-specific antibodies, neither by homologous, nor heterologous prime-boost regimens (Figure 5A). Surprisingly, the levels of serum M2e-specific IgG antibody, as well as IgG1 and IgG2a isotypes were significantly higher in mice from the homologous prime-boost group (H1N1+4M2e→H1N1+4M2e), compared to the heterologous prime-boost group (H1N1+4M2e→H3N2+4M2e), probably due to the better ability of the H1N1 subtype LAIVs to replicate in mouse respiratory tract than the H3N2 subtype LAIV viruses (Figure 5A). Nevertheless, high levels of H3N2-specific HAI antibody were observed in mice from heterologous prime-boost regimens, indicating that the second immunization with heterologous H3N2 LAIVs was successful (Figure 5B). Both homologous and heterologous prime-boost immunization regimens induced similar levels of H1N1-specific serum IgG antibody. As expected, higher levels of H3N2 whole virus binding IgG antibody were seen in the heterologous prime-boost group; however, significant reactivity of immune sera from the homologous prime-boost group with the H3N2 whole virus was also observed (Figure 5B). This could be due to the use of whole influenza virus as an antigen and IgG antibody binding to other than HA and NA proteins could be detected in the assay.

### 3.6. Long-Term Cross-Protection Afforded by Homologous and Heterologous Prime-Boost Immunization with LAIV+4M2e Vaccine Candidates

**Figure 6:**
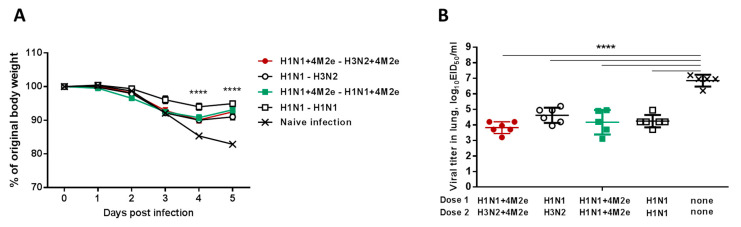
Protection against challenge with H5N1 virus of different LAIV immunization protocols. (**A**) Dynamics of body weight change. (**B**) Infectious viral titers in mouse lungs three days after challenge with rgH5N1 virus. Data were compared with two-way (**A**) or one-way (**B**) ANOVA followed by Tukey’s multiple comparisons test (**** *p* < 0.0001).

All immunized mice were subjected to an infection with heterosubtypic lethal rgH5N1 virus (40 LD_50_) 14 weeks after the second LAIV dose. Five days after the challenge, mice were sacrificed and different tissues were collected for virological and immunological analyses. Both homologous and heterologous vaccination regimens significantly protected mice against body weight loss (<10% and a sign of recovery by day 5 after challenge) and viral replication in lungs, compared to unvaccinated naïve mice after challenge infection (Figure 6A,B). The homologous prime-boost group (H1N1→H1N1) showed a less weight loss trend than heterologous (H1N1→H3N2) immunization after rgH5N1 virus challenge although the difference was not significant. The heterologous 4M2e group (H1N1+4M2e→H3N2+4M2e) displayed slightly lower lung viral titers than the heterologous LAIV group (H1N1→H3N2) but the differences were not significant among the LAIV-vaccinated groups; however, direct comparison of these groups by non-parametric Mann–Whitney test revealed significant difference (*p* = 0.026) (Figure 6B). These results suggest that homo and heterologous immunizations of mice with two doses of either LAIV or LAIV+4M2e vaccines significantly contribute to cross protection and that immunity other than serum IgG specific for M2e might be contributing to this protection.

### 3.7. Antibody Immune Responses in Immunized Mice after Challenge with Heterologous Influenza Virus

**Figure 7:**
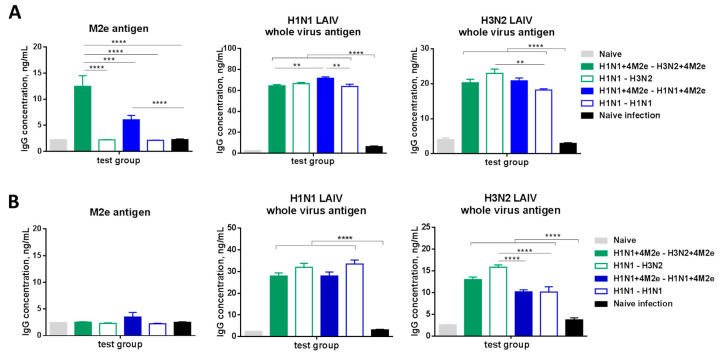
Antibody responses in mediastinal lymph nodes (**A**) and spleen (**B**) of immunized and naïve mice 5 days after infection with heterologous rgH5N1 challenge virus. Data were compared with one-way ANOVA followed by Tukey’s multiple comparisons test (** *p* < 0.01, *** *p* < 0.001, **** *p* < 0.0001).

A successful influenza vaccine should generate long-lived memory B cells that can rapidly differentiate into antibody-producing cells upon re-exposure to antigens [30]. To determine influenza virus-specific antibody secreting cell responses, cells from the mediastinal lymph nodes (MLN) and spleens were collected at day 5 after challenge and incubated in vitro for 5 days in 96 well plates coated either with hM2e peptide or with H1N1 LAIV, or with H3N2 LAIV inactivated viruses, followed by quantitative IgG ELISA. As seen in Figure 7A, significant levels of M2e-specific antibodies were secreted by MLN cells of mice immunized with chimeric LAIV+4M2e vaccines, in both homologous and heterologous prime-boost regimens. Noteworthy, the heterologous prime-boost vaccination protocol resulted in higher M2e-binding antibody production by MLN cells post-challenge, compared to the homologous prime-boost vaccination (Figure 7A). There was no secretion of M2e-specific antibodies detected in spleen cell cultures after the challenge (Figure 7B), suggesting that intranasal administration of the vaccine candidates resulted in generation of local memory B-cells which can rapidly respond to viral infection. As expected from H1N1 LAIV priming in all vaccine groups, higher levels of IgG antibodies specific for H1N1 viral antigens were secreted in in vitro cultures with MLN and spleen cells than those for H3N2 viral antigens (Figure 7A,B). These results support the development of local and systemic memory B cells to influenza virus antigens after homologous and heterologous prime-boost LAIV immunization. The induction of H3N2-specific IgG antibody even in the absence of this subtype LAIV in the homologous prime boost H1N1 immunization schedule suggests the activation and differentiation of B cells targeted to conserved epitopes distributed in various viral proteins.

### 3.8. Potential Contribution of Innate Immune Cells and T-Cell Responses to Protection in Immunized Mice after Challenge with Heterologous Influenza Virus

**Figure 8:**
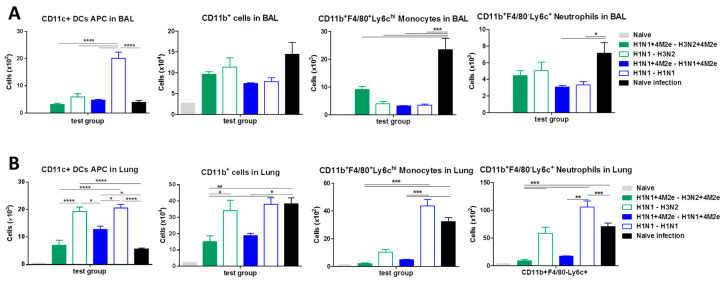
Dendritic and innate immune cells in the airway bronchoalveolar lavage (BAL) fluids (**A**) and lungs (**B**) in LAIV-vaccinated mice and naïve mice at day 5 after infection with heterologous challenge virus. Data were compared with one-way ANOVA followed by Tukey’s multiple comparisons test (* *p* < 0.05, ** *p* < 0.01, *** *p* < 0.001, **** *p* < 0.0001).

Innate immune cells are known to be recruited into the BAL fluids and lung due to inflammatory signals (e.g., cytokines) as a result of virus infection and are known to have both effects—inflammatory disease and generation of adaptive immunity in surviving hosts [31]. To understand the potential contribution of innate immune cells to protection or inflammation, we analyzed dendritic and innate immune cells in the airway BAL fluids and lungs in LAIV-vaccinated mice and naïve mice at day 5 after challenge infection with heterologous rgH5N1 virus (Figure 8). Flow cytometry data showed that CD11c^+^MHCII^+^ DCs and CD11b^+^ myeloid innate immune cells were recruited to the airway BAL fluids (Figure 8A) and lungs (Figure 8B) in LAIV-vaccinated mice, particularly in the homo prime boost H1N1 group at significantly higher levels than unvaccinated naïve mice after challenge infection. These results suggest that CD11c+ DCs recruited to the mucosal sites in LAIV-vaccinated mice might have contributed to inflammatory disease and generation of adaptive immunity in surviving hosts after challenge.

A different pattern of CD11b^+^ myeloid innate immune cells was observed in the airway BAL fluids and lungs. The highest levels of CD11b^+^ monocyte-derived macrophages were detected in the BAL fluids from naïve mice after infection, which might be associated with severe disease (Figure 8A). Moreover, the levels of CD11b^+^ monocytes and CD11b^+^ neutrophils were high in the lung from naïve mice after infection (Figure 8B). Among the LAIV-vaccinated mice, the levels of CD11b^+^ myeloid innate immune cells were significantly higher in the LAIV groups (H1N1→H1N1, H1N1→H3N2) than those in the LAIV+4M2e groups (Figure 8B). These results indicate that high levels of CD11b^+^ myeloid innate immune cells in the respiratory sites might be contributing to inflammation due to influenza virus infection.

**Figure 9:**
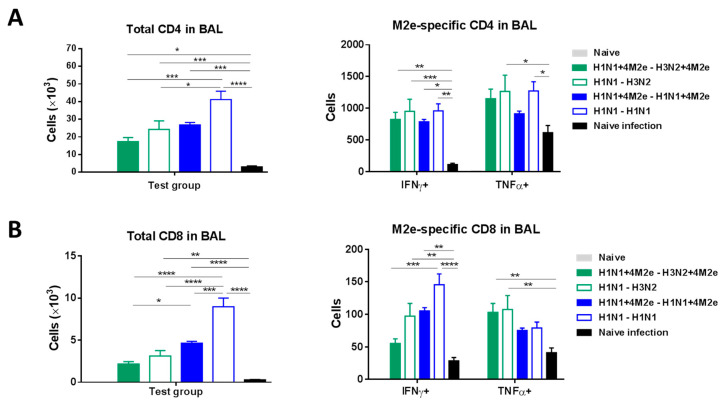
CD4 (**A**) and CD8 (**B**) T-cell responses in the airway BAL fluids of LAIV-vaccinated mice and naïve mice at day 5 after infection with heterologous rgH5N1 challenge virus. The left panel shows total number of cells in BAL. The right panel shows numbers of cytokine-producing cells after in vitro stimulation with human M2e peptide. Data were compared with one-way (left panel) or two-way (right panel) ANOVA followed by Tukey’s multiple comparisons test (* *p* < 0.05, ** *p* < 0.01, *** *p* < 0.001, **** *p* < 0.0001).

**Figure 10:**
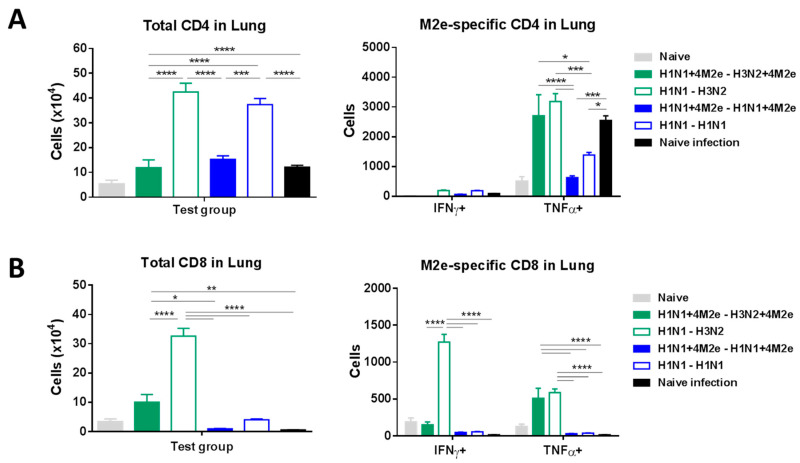
CD4 (**A**) and CD8 (**B**) T-cell responses in the lungs of LAIV-vaccinated mice and naïve mice at day 5 after infection with heterologous challenge virus. Left panel shows total number of cells in BAL. The right panel shows numbers of cytokine-producing cells after in vitro stimulation with human M2e peptide. Data were compared with one-way (left panel) or two-way (right panel) ANOVA followed by Tukey’s multiple comparisons test (* *p* < 0.05, ** *p* < 0.01, *** *p* < 0.001, **** *p* < 0.0001).

LAIV can induce T-cell responses contributing to broader protection [18]. Significantly higher levels of IFN-γ and TNF-α cytokine-secreting CD4 T cells were generated upon M2e in vitro stimulation of the airway BAL cells from all LAIV-vaccinated mice at day 5 post-challenge infection compared to naïve mice as determined by flow cytometry ICS analysis (Figure 9A). Similarly, IFN-γ and TNF-α cytokine secreting CD8 T cells were detected after M2e in vitro stimulation in BAL fluid samples from LAIV immunized mice after challenge (Figure 9B). Interestingly, two-dose vaccination with H1N1 LAIV induced the highest levels of CD4 and CD8 T cells which were rapidly recalled upon challenge with heterologous virus; however, no significant differences were observed in the proportion of M2e-specific T cells relative to the total amount of CD4 or CD8 T cells post-challenge between the LAIV or LAIV+4M2e groups (data not shown). A different pattern of CD4 and CD8 T-cell responses was observed in the lungs of rgH5N1-challenged mice: a heterologous prime-boost group (H1N1→H3N2) demonstrated the most pronounced recall responses, followed by homologous (H1N1→H1N1) group (Figure 10A,B). Nevertheless, the highest numbers of M2e-responsive CD8 T cells were seen in both heterologous prime-boost group (H1N1→H3N2 and H1N1+4M2e→H3N2+4M2e) (Figure 10B), whereas the highest levels of M2e-responsive CD4 T cells were seen in the H1N1+4M2e→H3N2+4M2e LAIV group, but not in the homologous group (H1N1→H1N1) (Figure 10A). These results suggest that different vaccination schedules can induce robust T-cell responses which are rapidly recalled upon challenge with a heterologous virus, but the reactivity of the induced T cells varies among the LAIV study groups. Overall, the M2e-specific T cells likely contributed to cross-protection in all LAIV groups, regardless of the expression of additional M2e epitopes within the HA molecule.

## 4. Discussions

In this study, we attempted to develop a universal influenza vaccine, i.e., the vaccine which would have a broader spectrum of protection than currently licensed seasonal influenza vaccines. Ectodomain of M2 protein (M2e) is one of the most promising conservative proteins of the influenza virus since it is highly conserved among all influenza A viruses circulating in human population since 1918. At the same time, M2e protein itself is a very poor immunogen; therefore, many different strategies have been explored to enhance its immunogenicity [7,32,33]. In our current study, we explored a new platform for the development of M2e-based vaccine—a live attenuated influenza vaccine technology. LAIV has been successfully used in Russia since 1987; therefore, extensive studies have been conducted on safety, as well as on its genetic stability. LAIV virus replicates in the epithelium of the upper respiratory tract and produces not only humoral, but also local (secretory), as well as cellular immunity in vaccinated people [18] which already may contribute to the protection of vaccinated people against drifted influenza viruses. M2 is a homotetramer consisting of two subunits linked by a disulfide bond, held together by covalent interactions [34], so an idea was to use M2e as a tetramer to form a compactly folded protein, thereby ensuring the correct geometry of the virus particles.

Previous studies reported different strategies to further enhance the cross protective efficacy by mimicking the current vaccine platforms. Recombinant reassortant influenza H1N1 virus A/Puerto Rico/8/1934 (A/PR8), a mouse-adapted laboratory strain wild type (WT), was engineered to express chimeric 4M2e-HA where tandem M2e epitopes were inserted into the N-terminus HA1 domain [22]. Moreover, inactivated recombinant WT A/PR8 viruses containing a chimeric HA with a single M2e epitope in the head domain was tested as a cross protective virus vaccine platform [35]. These recombinant WT A/PR8 viruses were pathogenic in mice, limiting the application of developing vaccines. In an attempt to improve cross-protective properties of LAIV, we modified its genome by inserting additional M2e epitopes at the N-terminus of the hemagglutinin HA1 subunit. Importantly, the insertion of four M2e tandem repeats did not alter replicative characteristics of the LAIV reassortant viruses, despite the expression of the correctly folded M2e proteins on the surface of viral particles, as was confirmed by ELISA with 14C2 monoclonal antibody. The replicative patterns of classical LAIV viruses and the LAIV+4M2e variants were identical both in vitro (eggs and MDCK cells) and in vivo (upper and lower respiratory tract of BALB/c mice). It should be noted that our attempts to rescue an LAIV virus expressing five tandem repeats in a chimeric hemagglutinin conjugate was unsuccessful (data not shown), suggesting that the LAIV genome might have a limited capacity for the incorporation of additional genetic material in its genes, especially in a case where these additional proteins are being incorporated into a live viral particle.

The insertion of four M2e tandem repeats into genome of LAIV viruses resulted in enhanced production of M2e-specific antibody; however, detectable levels were induced only after inoculating mice with two doses of the vaccine. Despite different levels of M2e-reactive antibody, all mice vaccinated either with LAIV or corresponding LAIV+4M2e variants were fully protected against lethal challenge with a panel of heterologous influenza A viruses. These data are in line with the findings of previous multiple studies that LAIVs can confer heterosubtypic protection [19,36,37,38]. This cross-protective potential of LAIVs has been attributed to the induction of broadly-reactive T cells. The significant impact of M2e immunity on conferring higher efficacy of cross protection might have been masked by induction of cross protective T cell immunity in control prime boost LAIV-vaccinated mice. Indeed, the protective role of the M2e-specific antibody was seen in a passive serum transfer experiment, where clear difference in the survival rates between classical H3N2 LAIV and the chimeric H3N2+4M2e LAIV was demonstrated for two heterologous challenge viruses.

We then attempted to enhance the M2e antibody responses by boosting H1N1+4M2e immunized mice with a heterologous recombinant virus H3N2+4M2e, expecting a decrease in the immunodominance of B-cell epitopes spanning the HA and NA molecules. Unexpectedly, the opposite effect was observed: the M2e-specific antibody levels were higher in the homologous than in the heterologous prime-boost group, which most probably results from the better replicative properties of the H1N1+4M2e LAIV strain, compared to the H3N2 LAIV+4M2e virus. Nevertheless, such heterologous prime-boost regimen (H1N1+4M2e→H3N2+4M2e) induced robust long-lived M2e-specific B-cells since high levels of M2e antibody were secreted by MLN cells collected 5 days post-challenge after their in vitro stimulation with M2e protein. These M2e-specific B cells were also induced by the homologous (H1N1+4M2e→H1N1+4M2e) vaccination regimen, but at a significantly lower level, which is in contrast to the serum M2e-specific antibody levels detected in mice 3 weeks after final vaccination. These disparate results can be partially explained by a divergent pathway of differentiation of M2e epitope-targeted B cells into a memory subset, which might be affected by the immunodominance of the HA protein. Furthermore, since the enhanced secretion of M2e-specific antibody was noted in the chimeric LAIV+4M2e groups only in MLN cells, but not in splenocytes, the site of LAIV administration might have a critical impact on the induction of long-lived B-cell repertoire targeting various viral epitopes [39]. One of the study limitations is that we did not assess functional activity of the induced M2e antibody, as Fc-mediated functions are known to possess protective potential when antibodies are non-neutralizing. Furthermore, due to the non-neutralizing nature of the M2e antibody, we did not assess the levels of M2e-specific secretory IgA antibodies which normally act by steric hindrance, whereas M2e protein within the viral particle is not readily accessible to antibodies [40].

In this study, we also assessed possible contribution of M2e repeats within the chimeric LAIVs to modulating innate immune responses upon delayed (14 weeks post-immunization) infection with lethal heterologous influenza virus. The higher levels of CD11c+ dendritic cells and CD11b+ myeloid innate immune cells (eosinophils, monocytes, neutrophils) in the classical LAIV groups than those in the LAIV+4M2e groups, without significant differences in virological and clinical outcomes of the infection between study groups further support the hypothesis on the dual roles of innate immune cells in the respiratory tract in protection and in causing inflammatory disease [41]. Another study limitation is that we did not monitor histopathological changes in the lungs of rgH5N1-challenged mice to fully elucidate the role of innate immune cells in the protection or inflammation.

As was noted above, immunization with live influenza vaccine viruses induces cross-protection in mice due to the induction of robust T-cell responses to multiple epitopes located throughout the viral proteome [18,22,42,43], which limits the use of this animal model to study specific T-cell epitopes within proteins of interest, such as M2e repeats. Immunodominance of some influenza virus epitopes might prevent the development of T-cell responses to less immunodominant epitopes [42,44], which could be one of the reasons that no significant increases in cross protective efficacy by M2e-specific immunity were observed in the LAIV+4M2e immunized mice, compared to the classical LAIV groups. Instead, more pronounced CD4 and CD8 T-cell responses were found in the classical LAIV rather than recombinant LAIV groups. A possible explanation for this could be the different replication kinetics of LAIV H1N1 and LAIV H1N1+4M2e given as the first dose in the mouse respiratory tract: although the kinetic was not monitored and viral titers were determined only at day 3 post inoculation, the differences in viral growth curves on MDCK cells support this suggestion. Another possibility is the impact of challenge virus replication on driving the induction of T cells. The H1N1-H3N2 group displayed a higher level of lung viral titers by approximately 10 folds after challenge with rgH5N1 virus. A group 1 and 2 HA virus prime boost strategy might differentially affect cross protective efficacy against the group 1 HA virus rgH5N1 challenge. Use of non-replicating vaccine platforms would provide an appropriate strategy for testing the efficacy of M2e immunity in mice. For example, different vaccine platforms which do not use live influenza virus were capable of inducing M2e-specific lung-resident memory CD4 and/or CD8 T cells, which correlated with the protection of mice against a lethal influenza virus infection [27,45].

## 5. Conclusions

As a proof-of-concept, we have developed recombinant influenza viruses expressing additional four M2e tandem repeats from the N-terminus of the viral HA protein, in an attempt to enhance the M2e-mediated cross-protection. Unlike similar constructs being assessed in animal models, the backbone used in this study was from a licensed live attenuated influenza vaccine—A/Leningrad/134/17/57, which underscores clinical relevance of the designed universal influenza vaccine candidates. Since LAIVs are inherently cross-protective, the elucidation of an additional protective role of the inserted M2e repeats in a mouse model is challenging. Nevertheless, our study suggests that M2e-specific antibodies can play a role in enhancing cross protection. The important finding is that the induced M2e-specific B-cell responses can be maintained for at least 12 weeks and rapidly recalled to produce antibody secreting plasma cells upon heterologous viral challenge. Overall, the new universal influenza vaccine candidates developed in this study warrant their further evaluation in relevant ferret preclinical studies toward a phase I clinical trial in volunteers.
